# Disease-linked mutations alter the stoichiometries of HCN-KCNE2 complexes

**DOI:** 10.1038/s41598-019-45592-3

**Published:** 2019-06-24

**Authors:** Yoann Lussier, Oliver Fürst, Eva Fortea, Marc Leclerc, Dimitri Priolo, Lena Moeller, Daniel G. Bichet, Rikard Blunck, Nazzareno D’Avanzo

**Affiliations:** 1 Department of Pharmacology and Physiology, Université de Montréal, Montréal, Canada; 2 Department of Physics, Université de Montréal, Montréal, Canada; 3 Department of Biochemistry and Molecular Medicine, Université de Montréal, Montréal, Canada

**Keywords:** Single-molecule biophysics, Supramolecular assembly

## Abstract

The four hyperpolarization-activated cylic-nucleotide gated (HCN) channel isoforms and their auxiliary subunit KCNE2 are important in the regulation of peripheral and central neuronal firing and the heartbeat. Disruption of their normal function has been implicated in cardiac arrhythmias, peripheral pain, and epilepsy. However, molecular details of the HCN-KCNE2 complexes are unknown. Using single-molecule subunit counting, we determined that the number of KCNE2 subunits in complex with the pore-forming subunits of human HCN channels differs with each HCN isoform and is dynamic with respect to concentration. These interactions can be altered by KCNE2 gene-variants with functional implications. The results provide an additional consideration necessary to understand heart rhythm, pain, and epileptic disorders.

## Introduction

The four mammalian homologs of hyperpolarization activated cyclic-nucleotide gated (HCN1–HCN4) channels represent the molecular correlate of the currents I_f_ or I_h_ in cardiomyocytes and neurons^1,2^. The sensitivity of HCN channels to cyclic nucleotides such as cAMP and cGMP enables I_h_ to adjust to stimulation of the autonomic nervous system. In the heart, I_h_ serves as the primary initiator for the diastolic depolarization of sinoatrial node (SAN) and atrioventricular node (AVN) action potentials. In murine models, deletion of the HCN4 gene results in embryonic lethality due to failed maturation of pacemaking cells^3^ whereas HCN4 conditionally deficient mice have a 70–80% reduction in SAN I_h_^4^. HCN1 deficient mice display congenital sinus node dysfunction with severely reduced cardiac output^5^. HCN2 deficient mice display mild sinus dysrhythmia at rest^6^. Genetic variants in HCN channels are linked to sinus node dysfunction^7–11^, atrial fibrillation^12,13^, ventricular tachycardia^14,15^, atrio-ventricular block^16,17^, Brugada syndrome^13,18^, sudden infant death syndrome^19,20^, and sudden unexpected death in epilepsy^21^. Non-pacemaking atrial and ventricular cardiomyocytes also express HCN channels, with an increase in I_h_ of ventricular myocytes reported in hypertrophy, ischemic cardiomyopathy and heart failure^22–24^.

HCN channels are expressed not only in the heart but also throughout the central and peripheral nervous systems. All four isoforms are expressed in the brain^25–27^ where they play a role in setting the resting membrane potential, dendritic integration, neuronal pacemaking, and establishing action potential threshold^28^. HCN1 knockout mice show impaired motor learning but enhanced spatial learning and memory^29,30^ and an enhanced susceptibility to kainic acid induced seizures^31^. HCN2 knockout mice present symptoms of absence epilepsy and tremoring^6^. Mutations in HCN1 and HCN2 have been identified in patients with genetic generalized epilepsy^32,33^, genetic epilepsy with febrile seizures plus (GEFS+)^32^, epileptic encephalopathy^34^, idiopathic generalized epilepsy^35^, hyperthermia-induced neuronal hyperexcitability and febrile seizures in children^36^. HCN1 and HCN2 are predominant in large and small sized primary afferent (sensory) neurons such as the dorsal root ganglion^37^, where they play a role in pain sensation. HCN2 knockout mice also fail to demonstrate neuropathic pain in response to mechanical or thermal stimuli^38^ suggesting that I_h_ drives action potential firing to initiate neuropathic pain in nociceptors. HCN isoforms are expressed differentially in the retina, where I_h_ plays a role in the shaping of retinal responses to light, including limiting retinal hyperpolarization encoding brightness^39^. HCN channels also play important roles for olfaction, with 10% of the juxtaglomerular cells strongly expressing HCN1^40^, and sour taste sensation^41^.

KCNEs are a family of single-helix transmembrane proteins with 5 known members that modulate the function of several ion channels^42^ including HCN channels^43–46^. HCN regulation appears specific to KCNE2, while KCNE1, KCNE3 and KCNE4 have no effect^44^. KCNE2 effects are also specific to the HCN isoform^43^. Expression patterns of KCNE2 in cardiac tissue resembles that of HCN channels, with highest levels in the SAN followed by conduction tissue and atria, with lowest levels in ventricular cells^46,47^. Moreover, endogenous HCN2 and KCNE2 co-immunoprecipate in canine SAN cells, and rat neonatal ventricular myocytes^45^. The mutation M54T in KCNE2 is genetically linked to sinus bradycardia and reduces I_h_ density in neonatal rat ventricular myocytes by 80% and slows activation kinetics at physiologically relevant voltages^48^. Increased HCN2, HCN4 and KCNE2 expression in ventricular myocytes may contribute to ventricular arrhythmogenesis after acute myocardial infarction^49^. KCNE2 mRNA is also found^50^ in brain regions that express HCN isoforms^51–53^. Targeted deletion of KCNE2 shifts the voltage dependence and alters I_h_ activation and deactivation kinetics in layer 6 pyramidal neurons, down-regulates HCN1 and HCN2 expression in the brain and results in hyper-susceptibility to the convulsant 4-AP^54^. These results indicate that HCN-KCNE2 complexes have important physiological implications in both cardiac and neuronal function. However, the molecular details of these complexes are not yet understood. Here, we studied how many regulatory KCNE2 subunits interact with the pore forming HCN subunits, and whether the stoichiometry is altered by genetic variations.

## Results

### KCNE2-HCN stoichiometry alters dynamically with relative expression

**Figure 1:**
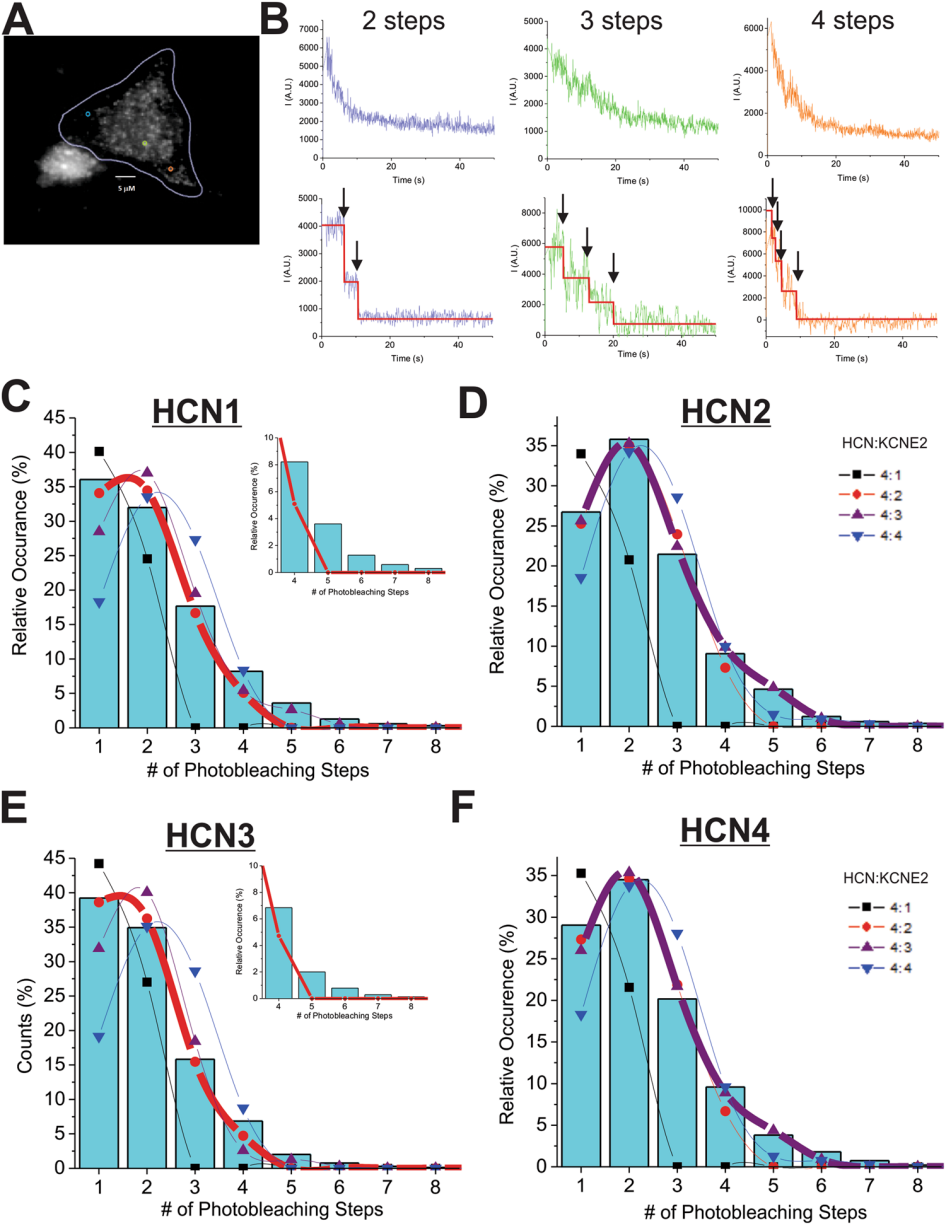
*PIF* automated subunit analysis of HCN-KCNE2 complexes. (**A**) Spots of interest are automatically selected from a user-defined region of interest (outlined in blue) that encloses the cell. (**B**) (*top*) The fluorescence intensities of three spots are shown as examples. (*Bottom*) Following the filtering and step detection algorithms, traces are idealized, assessed against 5 quality control criteria, are accepted/rejected, and then steps are counted. Distributions of KCNE2-msfGFP in complex with HCN1 (**C**), HCN2 (**D**), HCN3 (**E**) or HCN4 (**F**) expressed in CHO-K1 cells were fit to a “Poisson distribution of a binomial distribution” function of n^th^ order (1 ≤ n ≤ 4 KCNE2 subunits) (Eq. 1) that accounts for the maturation of msfGFP (0.55), the number of unique channel complexes, complexes containing only KCNE2, and complexes that overlap on the same spot. KCNE2 in complex with HCN1 and HCN3 channels are best fit with an 2^nd^ order function (2 KNCE2’s per complex), while HCN2 and HCN4 are best fit with a 3^rd^ order function (3 KCNE2’s per complex). This distribution did, however, have a large residual between the fits and the number of spots observed containing >4 photobleaching steps (*insets of C&E*). Colocalization parameters (p_col_) for the best fits where 0.41, 0.17, 0.37, and 0.17 for HCN1, HCN2, HCN3, and HCN4 respectively.

To assess the stoichiometry of the HCN-KCNE2 assembly, we used the single-molecule fluorescence subunit counting (SSC) technique^55^. In SSC, the stoichiometry is calculated from the number of photo-bleaching steps of a fluorescently-labelled protein (Fig. 1A,B). For our experiments, KCNE2-msfGFP fusion protein was co-expressed with HCN isoforms in Chinese hamster ovary (CHO-K1) cells in a 1:1 ratio (w:w) and imaged by total-internal reflection (TIRF) microscopy. The maturation probability of GFP (p_m_) depends exclusively on the expression system, the temperature during maturation, and the oxidation state but not the protein of interest^55^. It can therefore be determined in advance, using a protein of known stoichiometry, and subsequently kept fixed to reduce the number of free parameters. Here, we used AQP2-GFP (Supp. Fig. 1) whose tetrameric stoichiometry is known from crystal structures^56–59^. This enabled us to constrain the p_m_ to 0.55–0.56, similar to values determined in other studies^55,60^. Data collected from cells transfected with KCNE2-GFP + empty vector (Supp. Fig. 2) were used to account for any KCNE2 that traffics to the plasmalemma or to intracellular organelles within TIRF distance either on its own or in complex with endogenous proteins. This background KCNE2 expression attributed to less than 15% of the analyzed spots and was subtracted from the distribution collected in the presence of HCN channels.

We first analyzed the photobleaching step histogram for each HCN-KCNE2 complex using a binomial distribution of varying order up to 4 KCNE2 per HCN tetramer (Eq. 1). Based on our AQP2-GFP experiments (Supp. Fig. 1), the maturation probability of GFP was narrowly constrained to p_m_ = 0.55–0.56 whereas the probability of overlapping spots (p_c_ i.e. two independent HCN channels within a diffraction limited spot) was not restricted. The distribution determined using Eq. 1 is consistent with 2 KCNE2 subunits forming a complex with HCN1 or HCN3 channels and 3 KCNE2 subunits forming a complex with HCN2 or HCN4 (Fig. 1C–F). This data indicates that complex formation with KCNE2 differs between HCN isoforms. However, substantial residuals remain between the binomial fits of 4:2 HCN1:KCNE2 and HCN3:KCNE2 complexes and the number of events observed with greater than 4 photobleaching steps (Fig. 1C,E insets). Moreover, given the tetrameric assembly of HCN subunits^61^, it is difficult to intuitively reconcile how exactly 3 KCNE2 subunits but not 4 could stably complex with HCN2 or HCN4 channels. This suggests that our initial assumption that HCN-KCNE2 complexes are expressed with a fixed stoichiometry is not correct.

**Figure 2:**
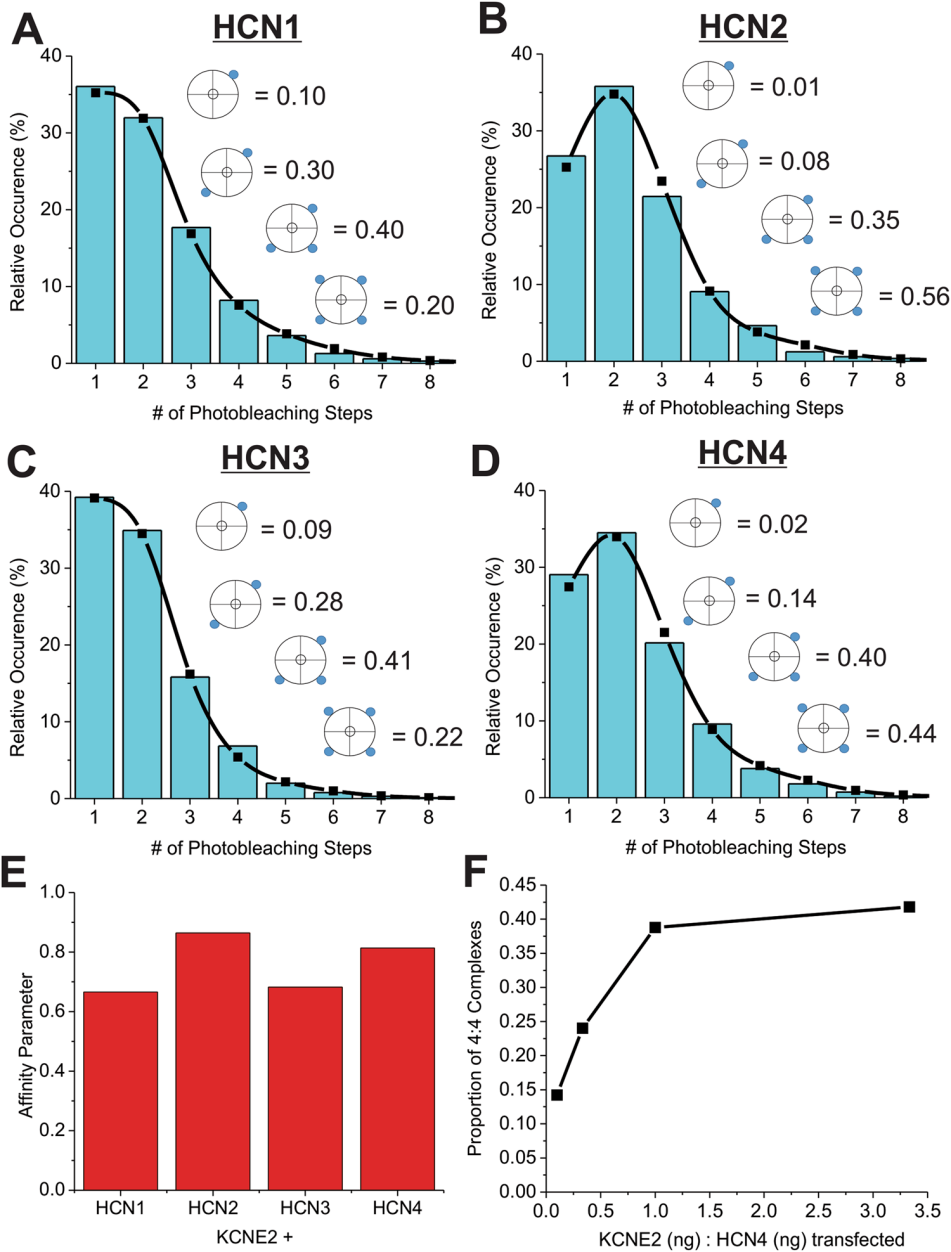
Photobleaching steps analysis using a linear superposition of distributions function. Step distributions of KCNE2-msfGFP in complex with HCN1 (**A**), HCN2 (**B**), HCN3 (**C**) or HCN4 (**D**) expressed in CHO-K1 cells were fit to a using a linear superposition of distributions that permitted a mix of complexes containing 1–4 KCNE2 subunits per HCN tetramer. The fraction of complexes for n = 1–4 KCNE2 subunits (blue circles) is also shown. (**E**) The affinity parameter from each fit is plotted for each HCN isoform. (**F**) We observe a concentration dependence of the 4:4 complex as we increase the ratio of HCN4:KCNE2 transfected into CHO-K1 cells.

One way to obtain a 3:4 stoichiometry would be that 4 KCNE2 binding sites exist but are not stably complexed but rather occupied with a specific affinity to the binding site. The occupancy would result in a superposition of 1,2,3 and 4 KCNEs per HCN dependent on the relative expression level. Accordingly, we re-analyzed the histograms using a linear superposition of distributions accounting for 1 to 4 KCNE2s per HCN tetramer (Eq. 2). In our model, the KCNE2s complex with HCN with equal affinity for each site (Fig. 2A–D). As expected, this algorithm predicts 100% of AQP2 spots examined were from tetrameric complexes. Fits of HCN-KCNE2 histograms using this model reduce the residual (especially in the region of events with >4 photobleaching steps) (Fig. 2A–D) and provided an affinity parameter (p_a_) for each complex (Fig. 2E) which in turn enables us to calculate the distribution of complexes containing 1–4 KCNE2 subunits. We observe the affinity parameter for HCN2 and HCN4 to be higher than HCN1 and HCN3 (Fig. 2E), suggesting these HCNs interact with KCNE2 subunits more favorably. Accordingly, the fraction of stoichiometries differed between isoforms, as well. 10% of HCN1 and HCN3 complexes contained only 1 KCNE2 while approximately 20% contained 4 KCNE2; these numbers shift to about 1–2% and approximately 50% for the HCN2 and HCN4 isoforms (Fig. 2A–D).

An affinity based model such as this predicts that the equilibria between complexes containing 1–4 KCNE2 subunits should depend on concentration of the constituents. To test this, we determined the number of photobleaching steps under varied transfected ratios of HCN4 and KCNE2 and analyzed the histograms of the different conditions. As predicted, we observed a concentration dependence to the proportion of 4:4 complexes calculated (Fig. 2F).

### Disease-linked mutations alter the function and assembly of HCN-KCNE2 complexes

**Figure 3:**
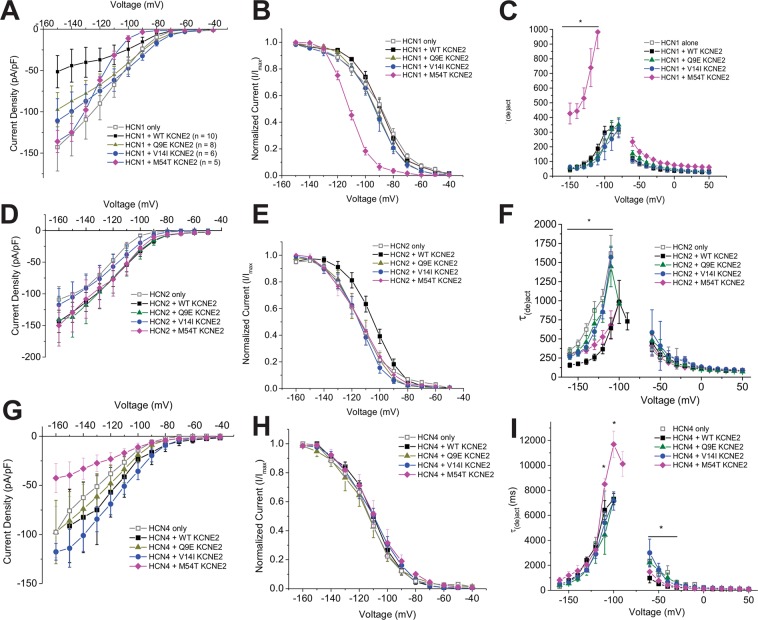
Effects of KCNE2 gene variants on HCN channel function determined by whole-cell patch clamp recordings. (**A**) Current-Voltage relationships (**B**) Steady-state activation curves and (**C**) voltage dependencies of activation and deactivation kinetics for HCN1 alone (*open square*; n = 10), HCN1 + WT KCNE2 (*closed squares*; n = 10), HCN1 + Q9E KCNE2 (*green triangles*; n = 8), and HCN1 + V14I (*blue circles*; n = 6). Similarly, (**D**) Current-Voltage relationships (**E**) Steady-state activation curves and (**F**) voltage dependencies of activation and deactivation kinetics for HCN2 alone (*open square*; n = 9), HCN2 + WT KCNE2 (*closed squares*; n = 10), HCN2 + Q9E KCNE2 (*green triangles*; n = 8), and HCN2 + V14I (*blue circles*; n = 9) were determined. Also, (**G**) Current-Voltage relationships (**H**) Steady-state activation curves and (**I**) voltage dependencies of activation and deactivation kinetics for HCN4 alone (*open square*; n = 8), HCN4 + WT KCNE2 (*closed squares*; n = 5), HCN4 + Q9E KCNE2 (*green triangles*; n = 5), and HCN4 + V14I (*blue circles*; n = 6).

Electrophysiology data indicate that KCNE2 expression has differing effects on the function of each HCN isoform. We observed that KCNE2 decreases the HCN1 current density in CHO-K1 cells with no change in the voltage-dependence of activation (HCN1 alone = −90.6 ± 0.8 mV vs HCN1 + WT KCNE2 = −89.0 ± 1.7 mV) or gating kinetics (Fig. 3A–C). Co-expression of WT KCNE2 has the largest impact on HCN2 channels. The voltage-dependence of activation is shifted by approximately + 8 mV, from −113.2 ± 2.5 mV to −105.0 ± 3.0 mV, and KCNE2 speeds the rate of HCN2 activation by approximately 3-fold (Fig. 3D–F). Co-expression of HCN4 with WT KCNE2 speeds the rate of deactivation by nearly 2-fold between −40 and −60 mV compared to HCN4 alone, with no effects on the voltage-dependence of activation (Fig. 3G–I; HCN4 alone = −113.8 ± 1.1 mV and HCN4 + WT KCNE2 = −110.2 ± 1.8 mV).

KCNE2-HCN complexes are relevant for cardiac and neuronal function^48,54^, suggesting that genetic variants of KCNE2 can modify their functional effects on HCN currents. Mutations in KCNE2 have been associated with congenital or drug-induced Long QT syndromes (LQT; Q9E, M54T)^62–67^, sinus bradycardia (Q9E, M54T)^48^ and sudden infant death syndrome (V14I)^20,68^. Genetic variants of HCN channels are also associated with cardiac arrhythmias^9–11,15,17,48,69^, epilepsy^32–36^, and sudden death^19^. Based on our discovery that HCN-KCNE2 complex stoichiometry differs between HCN isoforms, we hypothesized that KCNE2 mutants may also have differential effects on the function of different HCN isoforms. The KCNE2 M54T mutant for example has been shown to differentially affect HCN isoforms, altering HCN2 and HCN4 kinetics^48^. Thus, we examined the effects of three KCNE2 mutations, Q9E, V14I and M54T on HCN function. Whole-cell patch clamp experiments on CHO-K1 cells were performed on HCN1, HCN2 and HCN4 channels, which are the predominant isoforms expressed in the various regions of the brain and the heart’s conduction tissue.

In line with previous results^48^, we observed M54T KCNE2 has isoform specific effects on HCN channel function. M54T KCNE2 decreases the current density of HCN4 but not HCN2. Furthermore, M54T KCNE2 significantly slows activation kinetics of HCN4 channels at membrane potentials more depolarized than −110 mV (Fig. 3I). On the other hand, M54T KCNE2 slows activation kinetics of HCN2 compared to WT KCNE2 only at membrane potentials more hyperpolarized than −120 mV (Fig. 3F). The effects of M54T KCNE2 on the voltage-dependence of HCN2 and HCN4 activation (HCN2 + M54T KCNE2 = −116.3 ± 3.9 mV; HCN4 + M54T KCNE2 = −111.2 ± 4.3 mV) and deactivation kinetics are the same as those induced by WT KCNE2 (Fig. 3E,H). Intriguingly, M54T KCNE2 has drastic effects on HCN1 channels, not previously examined. M54T KCNE2 alters the I-V profile and increases the slope-conductance of HCN1 channels (Fig. 3A). The voltage-dependence of activation also shifts to more hyperpolarized potentials by over −20 mV (HCN1 + M54T KCNE2 −111.9 ± 0.9 mV) compared to HCN1 expressed with or without WT KCNE2 (Fig. 3B). Lastly, M54T KCNE2 slows HCN1 activation and deactivation kinetics by 2–3 times at all voltages (Fig. 3C).

Q9E and V14I KCNE2 variants also differentially alter the functional effects of KCNE2 regulation of each HCN isoform. Current densities of HCN1 in complex with Q9E or V14I KCNE2 subunits have current densities similar to HCN1 only channels. Both gene variants also do not affect voltage-dependence of activation or gating kinetics of HCN1 (Fig. 3A–C; Q9E = −93.1 ± 2.0 mV and V14I = −92.9 ± 2.7 mV). For HCN2, both Q9E and V14I KCNE2 mutations return the steady-state activation profile and activation kinetics to those of HCN2 alone (Fig. 3E,F; Q9E = −113.7 ± 4.3 mV and V14I = −116.6 ± 3.1 mV), with no effect on current density (Fig. 3D) or deactivation kinetics. Lastly, HCN4 co-expressed with Q9E or V14I KCNE2 subunits have similar gating kinetics and voltage-dependencies as HCN4 alone (Fig. 3G–I; Q9E = −112.8 ± 3.1 mV and V14I = −111.5 ± 2.7 mV).

**Figure 4:**
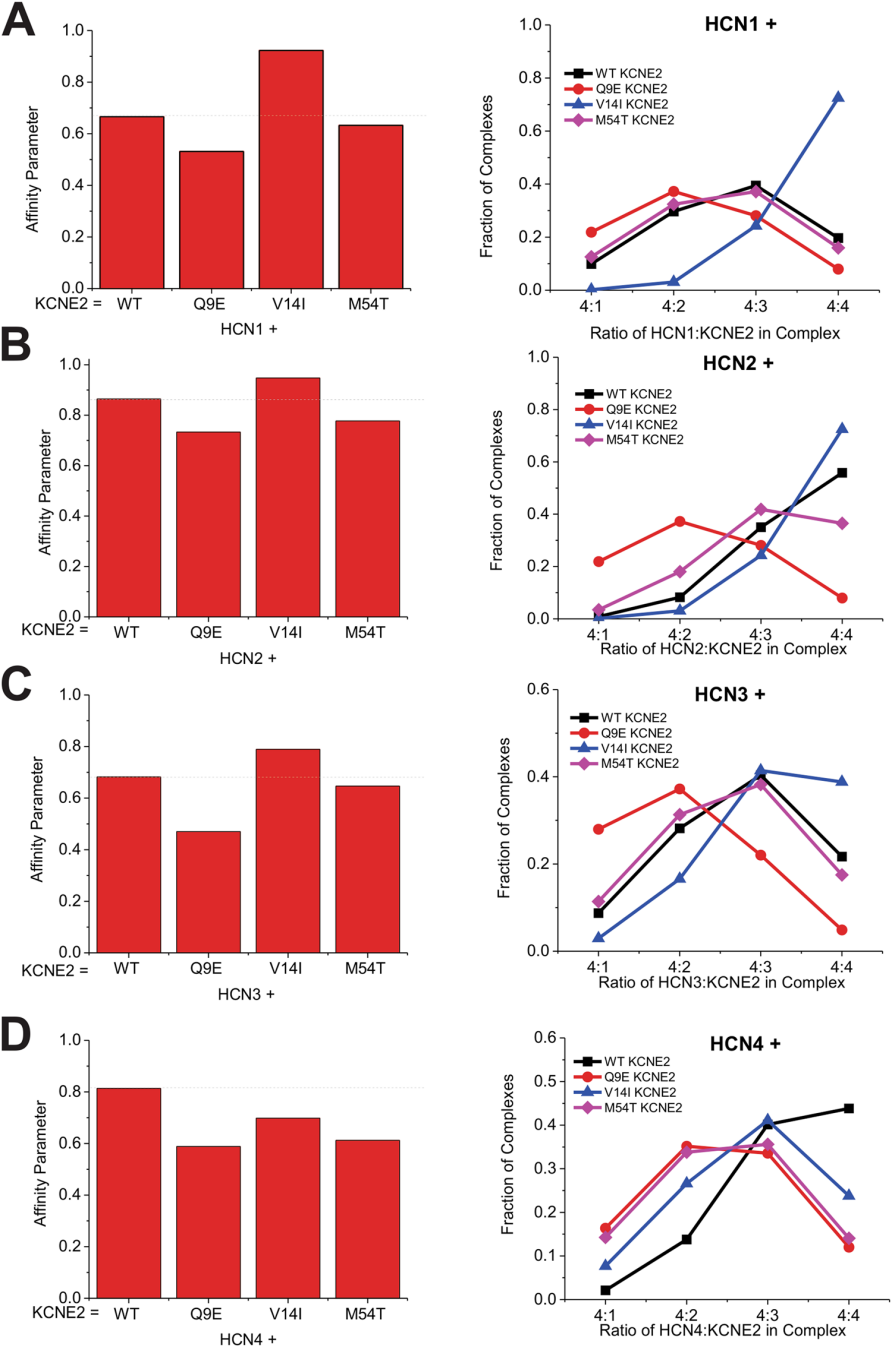
The effects of KCNE2 gene variants on HCN-KCNE2 stoichiometry. The binding affinity parameters (*left*) and fraction of 4:n HCN:KCNE2 complexes(*right*) we calculated from step distributions of KCNE2-msfGFP gene variants Q9E, V14I, and M54T in complex with HCN1 (**A**), HCN2 (**B**), HCN3 (**C**) or HCN4 (**D**). It is evident that these disease-linked KCNE2 mutations alter the stoichiometries within the HCN-KCNE2 complexes.

To assess if the differential functional effects of KCNE2 Q9E, V14I and M54T mutations on HCN isoforms is linked to their affinity for the channel subunits, we performed subunit-counting experiments for each HCN isoform co-expressed with these KCNE2 gene variants (Fig. 4 and Supp. Figs 2–6). KCNE2 M54T reduces the affinity for HCN2 and HCN4 subunits compared to WT, with a slightly larger effect on HCN4 channels. The difference in affinity of this mutant for HCN2 and HCN4 may explain the electrophysiological results previously reported^48^ where KCNE2 M54T reduces HCN4 current density and alters gating kinetics at physiologically relevant voltages while having a much weaker effect on HCN2 channels, with no change in current density and slower activation kinetics. However, the affinity parameter for M54T KCNE2 for HCN1 (and therefore the calculated distribution of HCN1- M54T KCNE2 stoichiometries) does not drastically differ compared to WT KCNE2. This suggests that gating parameters are not solely defined by the binding of KCNE2 subunits to HCN channels, and that the specific nature of the interactions contribute to the conformational changes necessary for gating. Q9E and V14I KCNE2 variants also had an impact on complex formation with each HCN isoform, with Q9E lowering KCNE2-HCN affinity in all cases, while V14I generally increased affinity except between KCNE2 V14I and HCN**4**. Therefore, our data indicates that disease-linked mutations in KCNE2 can alter the stoichiometry and function of I_h_ complexes in a manner consistent with an increase in susceptibility to cardiac arrhythmias and neuronal disorders such as epilepsy.

## Discussion

This study presents direct evidence of interactions between HCN channels and the auxiliary subunit KCNE2 using an unbiased single-molecule fluorescence assay^55^. We found that the simplest model of binding (a binomial distribution)—where HCN-KCNE2 complexes are formed with a fixed homogenous stoichiometry—could not properly account for the single subunit counting distributions collected from HCN1-KCNE2 and HCN3-KCNE2 complexes, particularly for observations of more than 4 photobleaching steps (Fig. 1C,E insets). Notably, a dimer of dimer model, whereby our data was fit with a mixture of complexes containing only 4:2 and 4:4 subunits also did not fit our data well. We therefore used a model with four identical binding sites for KCNE with equal binding affinity, leading to a dynamic distribution of up to 4 KCNE2s in complex with each HCN pore-forming unit (Fig. 2 and Supp. Figs 3–6). This model better described the collected distributions, as is evident by the reduced residual of fits. Moreover, our findings that the distribution of KCNE2 subunits in complex with HCN is concentration dependent (Fig. 2F) further supports this model. Our findings are similar to the proposed dynamic interactions between KCNQ1-KCNE1, which may vary between one to four KCNE1 subunits depending on concentration^70–73^.

The affinity parameter calculated from our linear superposition of distributions function (Eq. 2) of HCN1 and HCN3 are similar to one another, while HCN2 and HCN4 are more closely paired (Fig. 2F). Interestingly, the steady-state voltage-dependencies of these channels are similarly related, with HCN1 and HCN3 channels activated at more depolarized potentials than HCN2 and HCN4^74^. Moreover, if considering the core of the channel (S1–S6 + CNBD)—where KCNE2 is expected to interact—the phylogenetic relationship between HCN isoforms also follows this paired relationship between isoforms^75^. This suggests sequential differences in the core of the HCN channel isoforms are important for determining the binding affinity of complex formation with KCNE2.

Our results also indicate that KCNE2 subunits can traffic to the plasma membrane in the absence of HCN channels (Supp. Fig. 2). Similar results have been reported for KCNE1-mEGFP^70^ and suggested for KCNE2^76–78^. Since the distribution of KCNE2 alone was subtracted from our HCN-KCNE2 histograms prior to analysis, their presence do not affect the interpretation of our results, especially with respect to the implications of disease-linked mutations on complex stoichiometry. However, we should consider why KCNE is reaching the membrane at all. In this context, we should first consider how KCNE and HCN complexes are assembled. It is possible that free KCNE2 is present in the plasma membrane, which can then dock to the vacant sites present on HCN channels. Support for this model is provided from experiments where KCNE1 or KCNE3-containing lipid vesicles were delivered to the plasma membrane of *Xenopus* oocyte and modulated previously expressed KCNQ1 channels^79^. Alternatively, KCNE2 may co-assemble with HCN or Kv channels prior to plasma membrane expression forming stable stoichiometries lasting the lifetime of the channel. If KCNE2 cannot traffic by itself to the membrane, any KCNE2s present at the plasma membrane in the absence of co-expressed channels are likely interacting with errant native channels^80^. In our analysis, we corrected for these occurrences.

Our results also demonstrate that gene variants of KCNE2 that have been identified in patient populations can alter the stoichiometries of specific HCN isoform complexes with functional implications on I_h_. For example, the KCNE2 M54T mutation shifts the distribution of KCNE2 subunits found in complex with HCN4 and to a lesser degree HCN2. This mutation, which is genetically linked to sinus bradycardia, reduces I_h_ density in neonatal rat ventricular myocytes by 80% and slows activation kinetics at physiologically relevant voltages with preferential effects on HCN4 channels compared to HCN2^48^. We hypothesized that other KCNE2 mutants also have differential effects on HCN isoforms and that the specificity is linked to the affinity parameter. We therefore examined Q9E and V14I KCNE2 mutations and observed that they both eliminate the effects of KCNE2 on the HCN1,2, and 4 isoforms (Fig. 3) despite having differently affecting the subunit stoichiometry of the HCN-KCNE2 complexes (Fig. 4). This suggests that the simple interaction of KCNE2 subunits with the HCN isoforms is not itself sufficient to induce its functional effects. Rather it appears that the specific molecular details of the interaction between KCNE2 and HCN subunits are critical for instigating the functional changes that we and others have observed. Therefore, mutations such as Q9E and V14I appear to alter both the stoichiometries of the HCN-KCNE2 complexes, as well as the specific molecular interactions necessary for KCNE2 to induce functional changes in I_h_.

It is important to note a limitation to our assumption of linear superposition of distributions with equal affinity for each site in Eq. 2. It is notable in Fig. 2F, that the quantity of HCN complexes containing 4 KCNE2 subunits appears to saturate at approximately 40%. We speculate that this may be indicative of more complex binding behaviour (such as negative co-cooperativity between subunits) than the simple linear supposition of distributions that we used. However, if we try to fit our data with a model of this type of complexity, the degrees of freedom would increase and the reliability of any affinity parameters we estimate would drastically decrease. That said, several underlying trends that we observe would still remain: (1) that HCN:KCNE2 complexes form with a dynamic distribution rather than fixed stoichiometries, (2) that in the range we examined, the distribution of complexes formed with HCN1 and HCN3 are biased toward containing fewer KCNE2s than those formed with HCN2 or HCN4, and (3) KCNE2 gene variants would still alter the distribution of complex formation, in an HCN isoform dependent manner. Thus, given the data we are able to collect, the model of Eq. 2 still provides an accurate assessment of the dynamic behaviour of HCN-KCNE2 complexes and an excellent estimate of differences in affinity between HCN isoforms and different KCNE2 gene variants.

Altered I_h_ contributes to a variety of cardiac^7–18,22–24,69,81–85^ and neuronal disorders^32–36,38^. While the promiscuous nature of KCNE2 complicates the ability to establish a direct role for HCN-KCNE2 interactions in the development of these disorders there is ample evidence to support key roles for HCN-KCNE2 complexes in establishing the susceptibility to these disorders. The expression patterns of KCNE2 in cardiac and neuronal tissue resemble that of HCN channels^45–47,53,65^ and gene variants increase the susceptibility to several of the same cardiac and neuronal disorders linked to HCN such as epilepsy and cardiac arrhythmias^48,62–66,86,87^. Targeted deletion of KCNE2 alters the voltage dependence and kinetics I_h_ in neurons, down-regulates HCN1 and HCN2 expression in the brain and results in hyper-susceptibility to the convulsant 4-AP^54^. Also, copy number variants in KCNE2 were found in patients with schizophrenia^88^, a disorder also associated with increased HCN activity^89–91^. In addition, I_h_ has been shown to play important roles in motor learning, spatial learning and memory, sleep-wake cycles, and sensitivity to pain^29,30,38^. Since, KCNE2 gene variants are also present in 1.6–6% of the population^62,63^, differences in HCN-KCNE2 complexes may also contribute to the broad spectrum of these properties that exist in the general population.

## Methods

### Plasmid construction

All cDNAs were subcloned in pcDNA3.1(+) plasmid. KCNE2-sfGFP wild-type was added in-frame between HindIII/EcoRI cut sites, and the V206K mutation was added to reduce dimerization of the sfGFP. Subsequently, Q9E, V14I, and M54T mutations were introduced by PCR mutagenesis and verified by sequencing. Non-fluorescent or “dark” sfGFP was generated by introducing the G67V mutation into the chromophore of the GFP.

### Cell culture and transfection

Chinese hamster ovary cells (CHO-K1) were grown in Ham’s F12 Medium (Sigma) supplemented with 10% Fetal bovine serum (*Sigma*) and 1000 units of Penicillin-Streptomycin solution (Sigma) at 37 °C in a 5% CO_2_ environment. 24 h prior to transfections, cells were seeded into glass-bottom dish (MatTek Corp.) (McGuire, 2012) and transfected following Lipofectamine 2000’s (Invitrogen) protocol at 60–80% confluency. For patch-clamp experiments, cells were transfected with 2 µg HCN plus 2 µg of KCNE2 and 750 ng of GFP. For single-molecule subunit counting experiments, 10–100 ng of vector containing GFP-tagged or untagged versions of HCNs and KCNEs were used as listed. After 16–24 h, cells were washed 3 times with PBS at room temperature and fixed with EM-grade 4% formaldehyde (Ladd Research) in PBS for 24–36 h at 4 °C.

### Electrophysiology

Whole-cell patch clamp experiments were performed on CHO-K1 cells transfected with HCN1, HCN2, or HCN4 channels co-expressed of KCNE2 variants, 24–48 hours post-transfection. Glass pipettes with a final resistance of 2–2.5MΩ were used. The external and internal solutions contained (in mM): 150 KCl, 10 HEPES pH 7.3, 2 MgCl_2_ and 1 EGTA. All recordings were performed after 2 mins of dialyzing the internal solution following membrane rupture in order to avoid complications of current rundown. Data were collected at 22–25 °C at 10 kHz with a 1 kHz low-pass Bessel filter using a conventional Axopatch 200B Amplifier and Digidata 1440 A digitizer (Molecular Devices). Capacitance and series resistance were electronically compensated. HCN channel activation was assessed by eliciting voltage steps between −160mV and −40mV (Δ + 10 mV) from a holding potential (V_H_) of 0 mV, followed by a step to + 30 mV. Steady-state activation curves were determined from the peak of the tail currents. Deactivation was assessed by a pre-pulse to −130 mV followed by test pulses from +50 mV to −60 mV (Δ − 10mV). All protocols utilized a 17–24 s interpulse interval at V_H_ to ensure complete channel deactivation between test pulses. Data were analyzed using pClamp 10 (Molecular Devices) and Origin8.0 (OriginLab) software packages.

### Imaging

Fluorescent spots were recorded in TIRF configuration as previously described^55^. After washing the fixed sample in PBS, it was quenched by 700 microwatts of 488nm-laser light and collected at a sampling rate of 20 Hz on an EMCCD camera (iXon + 860BV, Andor Technology, South Windsor, CT).

### Single-molecule GFP analysis

The videos of photobleaching were processed using the *Progressive Idealization and Filtering* (PIF) software created by the Blunck laboratory^55^ where *min*. *Amplitude* was set at 1000, *dfof* at 10%, *Min Step Amp* at 800, *A*. *max step* at 4800 and all other parameters were left unchanged.

### Step distribution analysis

The step distribution was obtained by PIF^55^, normalized and analyzed using a binomial distribution when assessing the stoichiometry of AQP2 and the initial distributions for HCN-KCNE complexes:

1 $$P(N,k)=(1-{p}_{col})\cdot (\begin{array}{c}N\\ k\end{array}){p}_{m}^{k}\cdot {(1-{p}_{m})}^{N-k}+{p}_{col}\cdot (\begin{array}{c}2N\\ k\end{array}){p}_{m}^{k}\cdot {(1-{p}_{m})}^{2N-k}$$

where N represents the number of subunits present, k the number of detected steps, p_m_ is the probability of GFP maturation (ie. proportion of GFP that are fluorescent and can be photobleached), and p_col_ the probability of finding two spots at the same position (two channels within a diffraction limited spot).

To determine the affinity parameter, we assumed that the channel contains 4 binding sites each with an affinity (occupancy probability) p_o_. The distribution was then fitted according to:

2 $$P(k)=\sum _{i=1}^{4}(\begin{array}{c}4\\ i\end{array}){p}_{o}^{i}{(1-{p}_{o})}^{4-i}\cdot P(i,k)$$

where i represents how many binding sites are occupied.

## Supplementary information

Supplementary Figures

## Data Availability

The datasets generated during and/or analysed during the current study are available from the corresponding author on reasonable request.
