# Supplementary material for: Disease-linked mutations alter the stoichiometries of HCN-KCNE2 complexes
Source: Sci Rep. 2019 Jun 24;9:9113. doi: 10.1038/s41598-019-45592-3 (PMC6591248; doi:10.1038/s41598-019-45592-3)
Supplement: Supplementary file 1 — Supplementary Figures [file 41598_2019_45592_MOESM1_ESM.docx]

Disease-linked mutations alter the stoichiometries of HCN-KCNE2 complexes

**Authors:** Yoann Lussier^1^, Oliver Fürst^1^, Eva Fortea^1^, Marc Leclerc^1^, Dimitri Priolo^2^, Lena Möller^3^, Daniel G. Bichet^1^, Rikard Blunck^1,2^, Nazzareno D’Avanzo^1,3,*^

**Affiliations:**

^1^ Department of Pharmacology and Physiology, Université de Montréal, Montréal, Canada.

^2^Department of Physics, Université de Montréal, Montréal, Canada.

^3^Department of Biochemistry and Molecular Medicine, Université de Montréal, Montréal, Canada.

*Correspondence to: nazzareno.d.avanzo@umontreal.ca


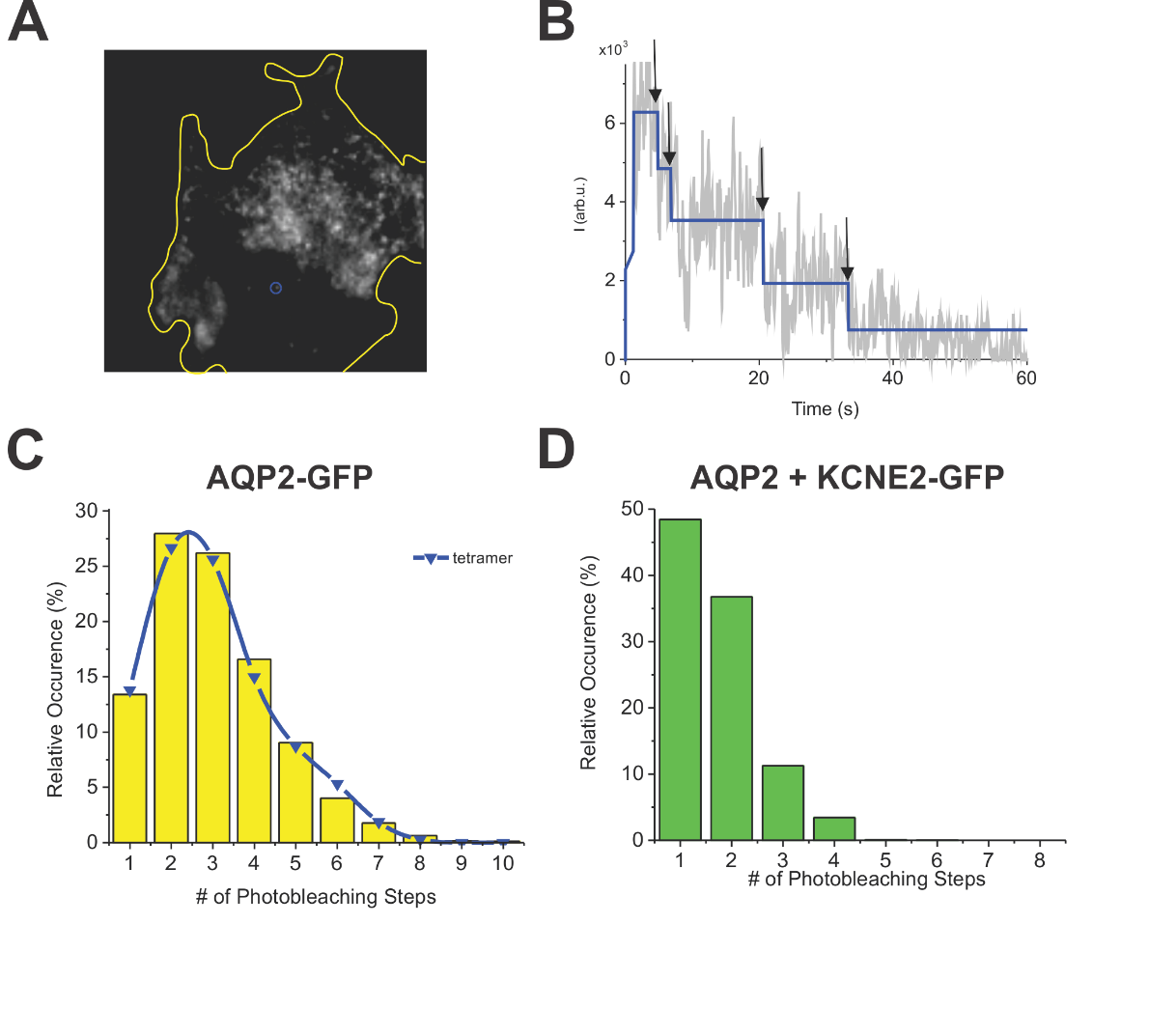


**Supp. Fig. 1. Calculation of GFP probability of maturation in CHO-K1 cells using aquaporin-2 (AQP2).**

Spots of interest are automatically selected from a user-defined region of interest (outlined in yellow) that encloses the cell using *PIF* software. **(B)** The fluorescence intensities in time (grey) are idealized (blue) and step detection algorithms are applied to generate histograms of photobleaching steps as in **(C)** (n = 4150). The histogram was fit with a binomial distribution function (Eq. 1) to assess the maturation of GFP (p_m_ = 0.55) which was then used as a restriction in fits of HCN-KCNE2 complexes. P_col_ = 0.44 and gives rise to the number of photobleaching steps above 4. **(D)** When KCNE2-subunits co-expressed with AQP2 channels, the distribution of photobleaching steps shits leftwards, and resembles more closely the distribution of KCNE2 subunits expressed alone (Supp. Fig. 2).

**
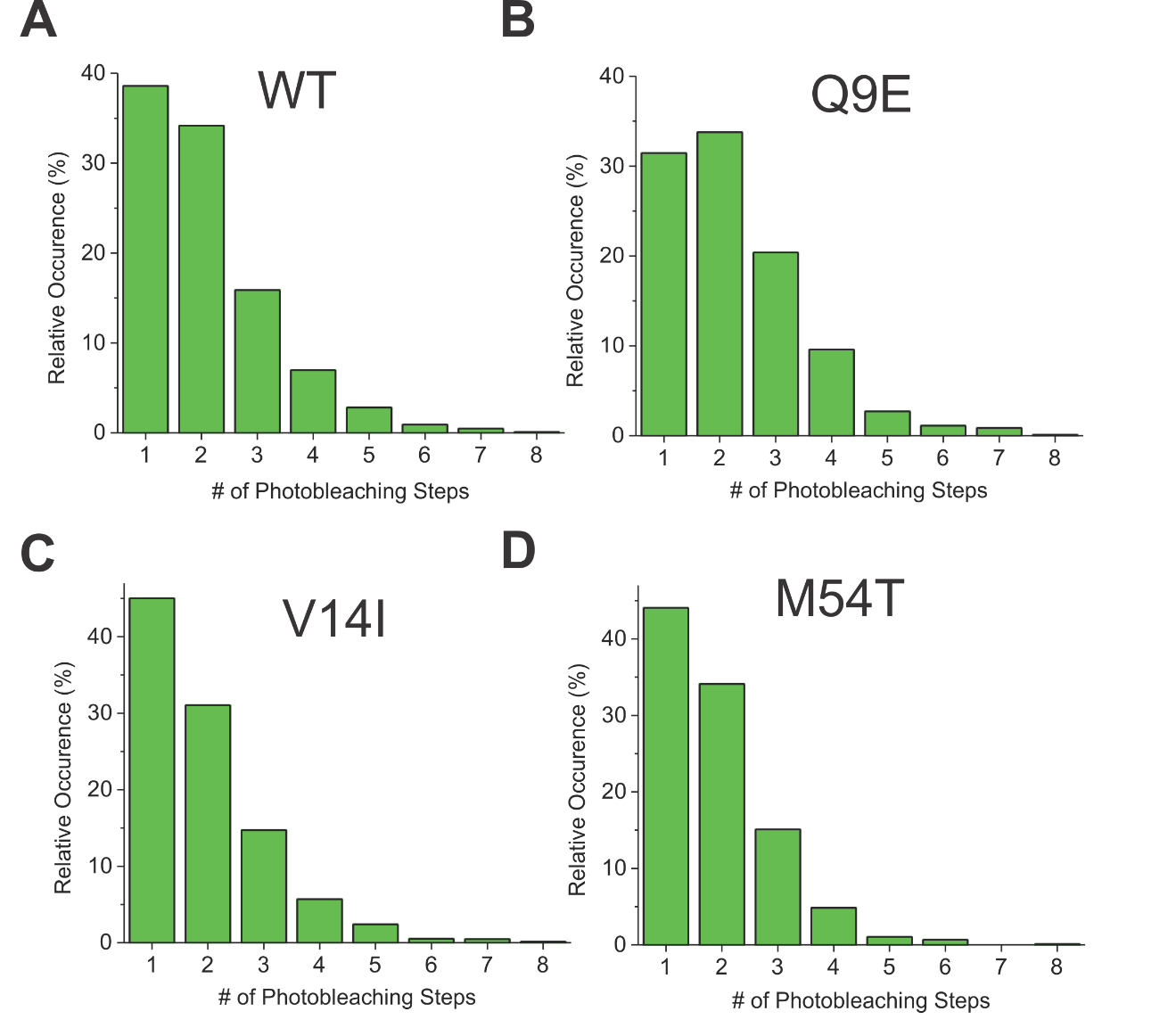
**

**Supp. Fig. 2. *PIF* automated subunit counting for WT and mutant KCNE2 proteins expressed in the absence of HCN channels.**

Step distributions of KCNE2-msfGFP expressed in the absence of HCN channels, for **(A)** WT (n=3294), **(B)** Q9E (n=814), **(C)** V14I (n=1584), and **(D)** M54T (n=1053) KCNE2-msfGFP variants.


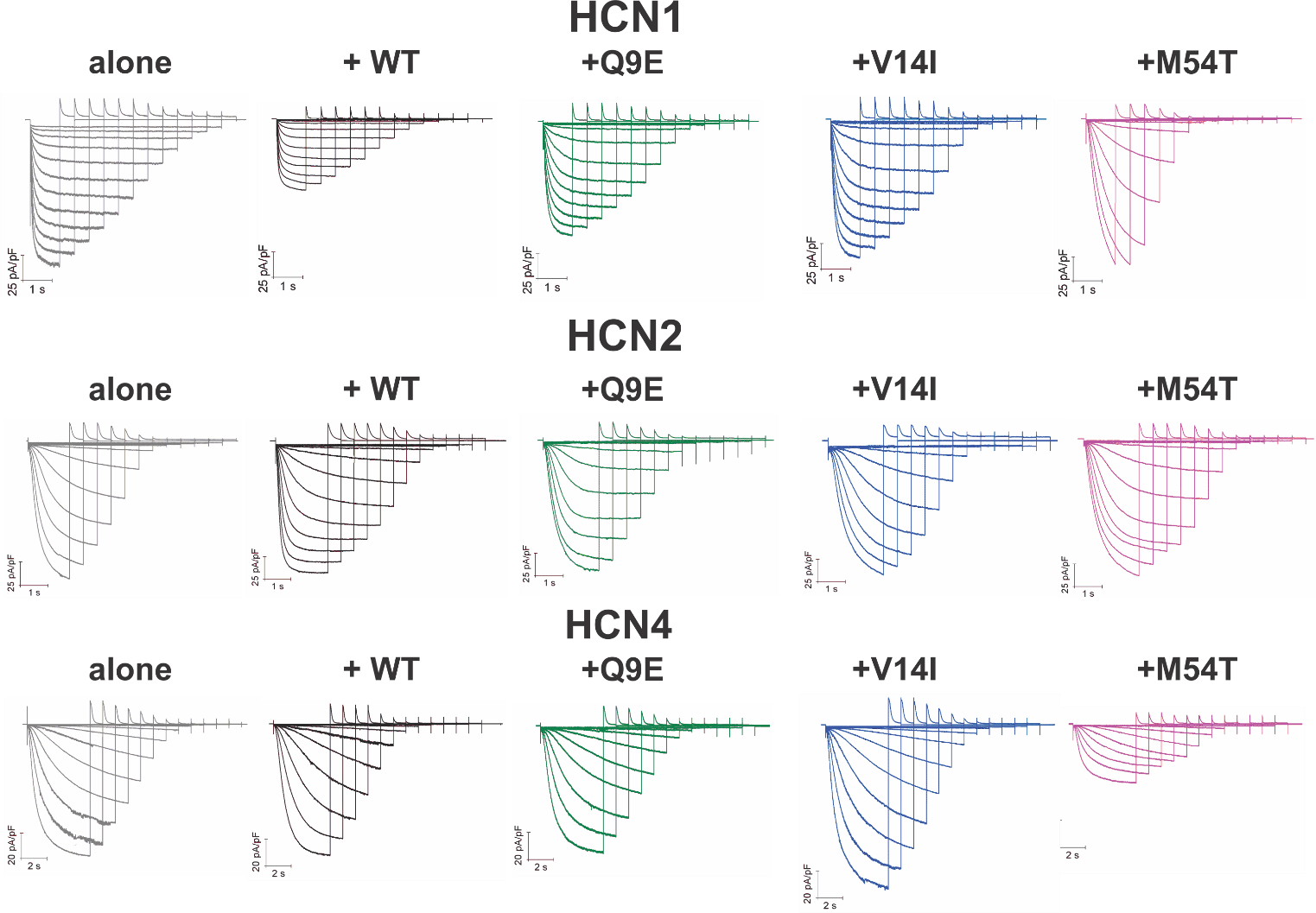


**Supp. Fig. 3. HCN1-KCNE2 subunit stoichiometry for WT and mutant KCNE2 subunits.**

Step distributions of HCN1 with **(A)** WT (n=3739), **(B)** Q9E (n=898), **(C)** V14I (n=1715), and **(D)** M54T (n=810) KCNE2-msfGFP variants expressed in CHO-K1 cells. Histograms were fit to using a a linear superposition of distributions that permitted a mix of complexes containing 1-4 KCNE2 subunits per HCN1 tetramer and the fraction of channels with 1 – 4 KCNE2 subunits was calculated from the affinity parameter.


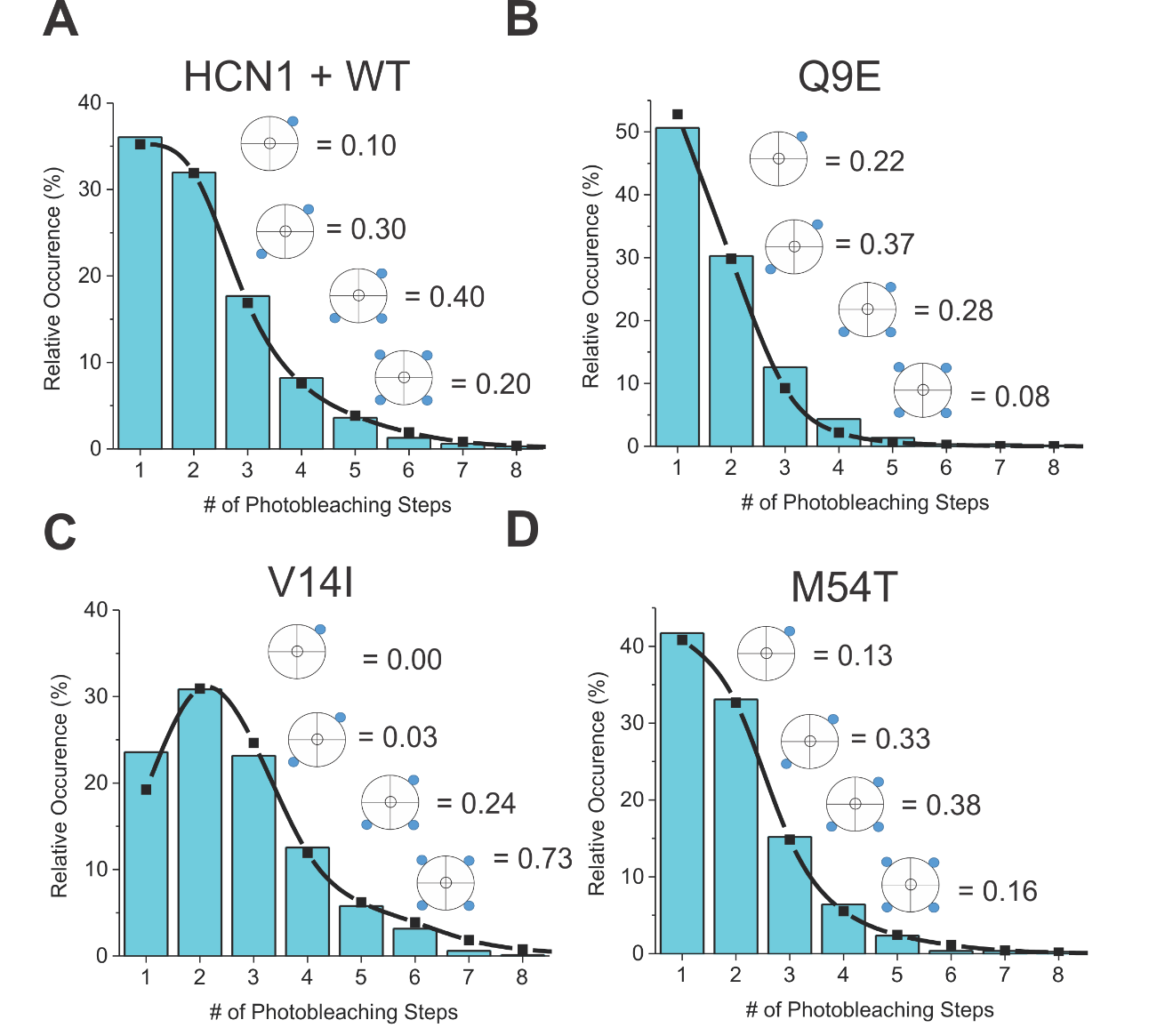


**Supp. Fig. 4. HCN1-KCNE2 subunit stoichiometry for WT and mutant KCNE2 subunits.**

Step distributions of HCN1 with **(A)** WT (n=3739), **(B)** Q9E (n=898), **(C)** V14I (n=1715), and **(D)** M54T (n=810) KCNE2-msfGFP variants expressed in CHO-K1 cells. Histograms were fit to using a a linear superposition of distributions that permitted a mix of complexes containing 1-4 KCNE2 subunits per HCN1 tetramer and the fraction of channels with 1 – 4 KCNE2 subunits was calculated from the affinity parameter.


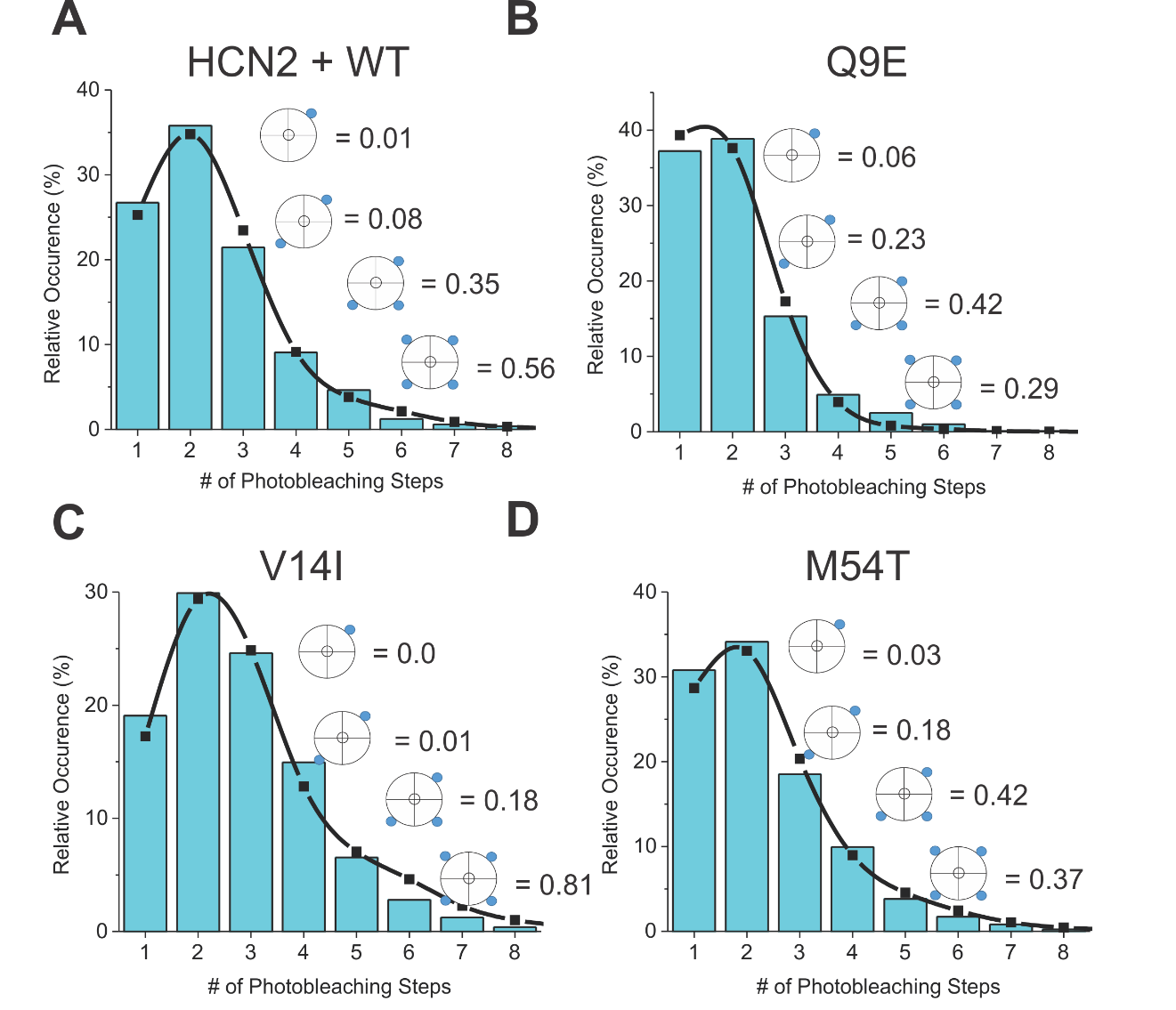


**Supp. Fig. 5. HCN2-KCNE2 subunit stoichiometry for WT and mutant KCNE2 subunits.**

Step distributions of HCN2 with **(A)** WT (n=3252), **(B)** Q9E (n=999), **(C)** V14I (n=1284), and **(D)** M54T (n=865) KCNE2-msfGFP variants expressed in CHO-K1 cells. Histograms were fit to using a a linear superposition of distributions that permitted a mix of complexes containing 1-4 KCNE2 subunits per HCN2 tetramer and the fraction of channels with 1 – 4 KCNE2 subunits was calculated from the affinity parameter.


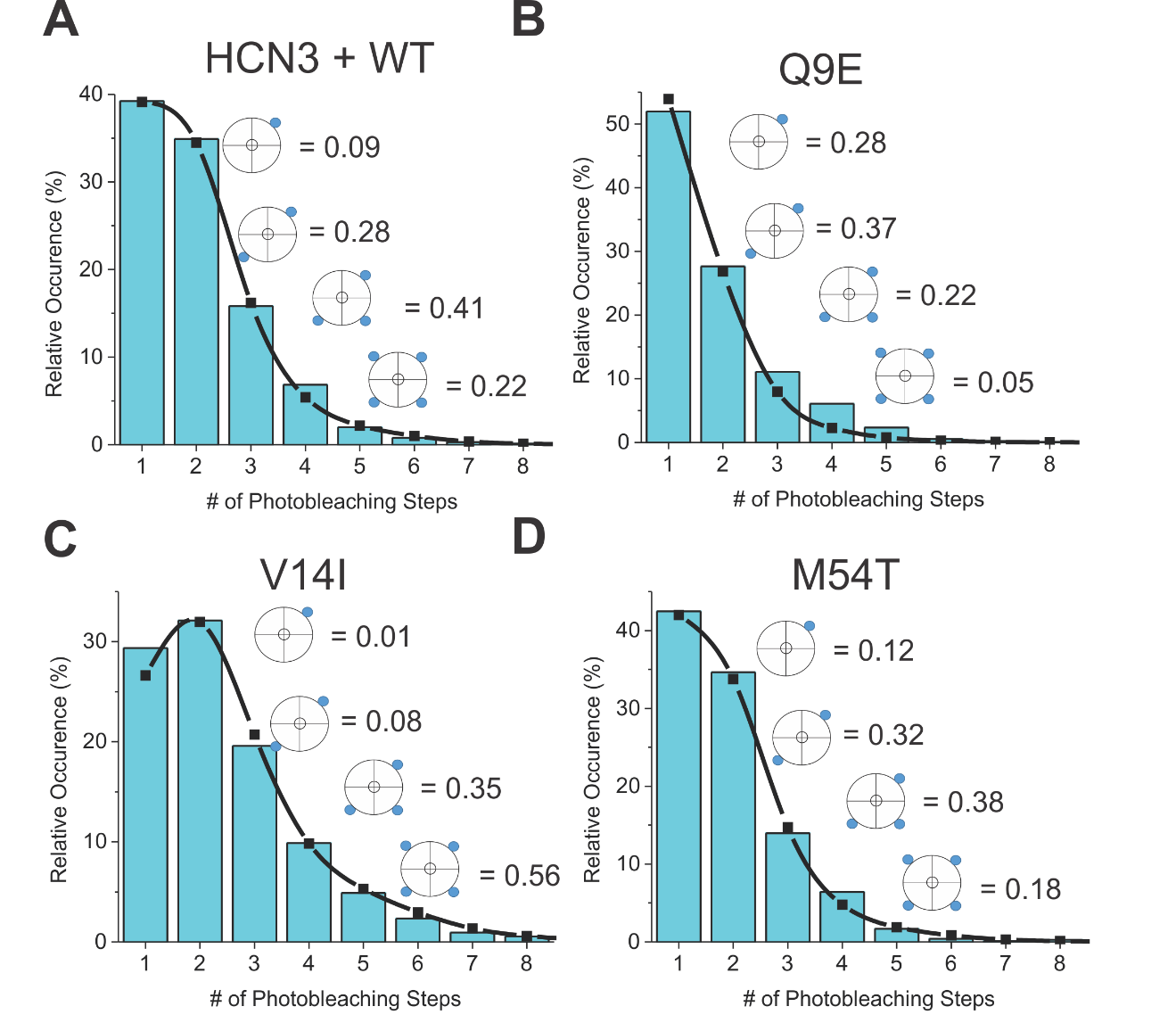


**Supp. Fig. 6. HCN3-KCNE2 subunit stoichiometry for WT and mutant KCNE2 subunits.**

Step distributions of HCN3 with **(A)** WT (n=2440), **(B)** Q9E (n=938), **(C)** V14I (n=1062), and **(D)** M54T (n=716) KCNE2-msfGFP variants expressed in CHO-K1 cells. Histograms were fit to using a a linear superposition of distributions that permitted a mix of complexes containing 1-4 KCNE2 subunits per HCN3 tetramer and the fraction of channels with 1 – 4 KCNE2 subunits was calculated from the affinity parameter.


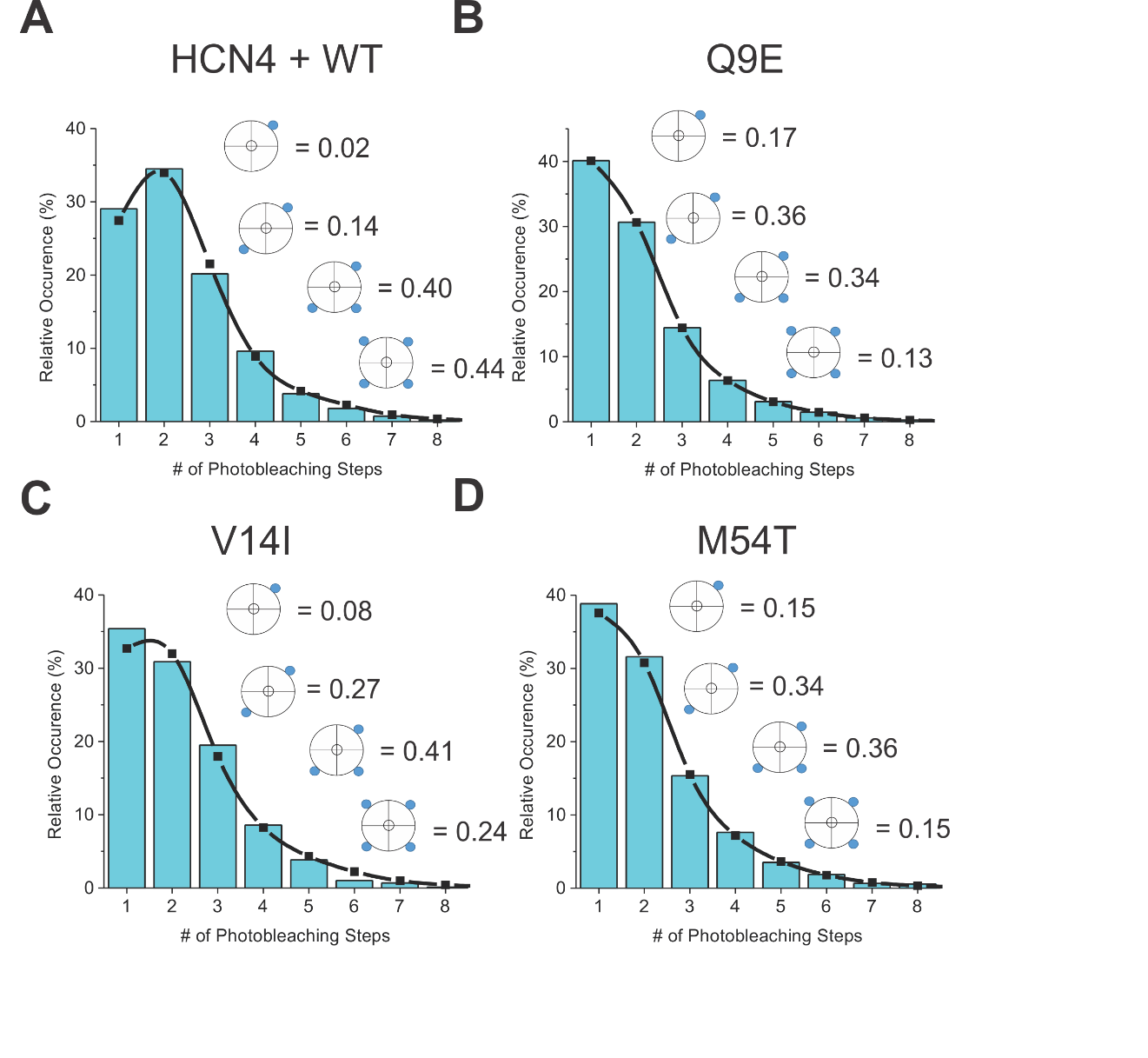


**Supp. Fig. 7. HCN4-KCNE2 subunit stoichiometry for WT and mutant KCNE2 subunits.**

Step distributions of HCN4 with **(A)** WT (n=3392), **(B)** Q9E (n=831), **(C)** V14I (n=887), and **(D)** M54T (n=1053) KCNE2-msfGFP variants expressed in CHO-K1 cells. Histograms were fit to using a a linear superposition of distributions that permitted a mix of complexes containing 1-4 KCNE2 subunits per HCN4 tetramer and the fraction of channels with 1 – 4 KCNE2 subunits was calculated from the affinity parameter.
